# Exploring the Use of Digital Technology to Support Health Behavior Change in Young People Under the Care of Complications of Excess Weight (CCEW) Clinics: Qualitative Patient-Centered Design Study

**DOI:** 10.2196/64947

**Published:** 2025-10-15

**Authors:** Brioney Gee, Bonnie Teague, Matt Farrar, Victoria Farrar, Dorothy Szinay, Li F Chan, Ken K Ong, Ben Jackson, Sydney Wylie, Felix Naughton, Jon Wilson, Emma Alice Webb

**Affiliations:** 1 Norfolk and Suffolk NHS Foundation Trust Research Norfolk and Suffolk NHS Foundation Trust Norwich United Kingdom; 2 Norwich Medical School University of East Anglia Norwich United Kingdom; 3 LovedBy Group Cheshire Loved By Group Chester United Kingdom; 4 School of Health Sciences University of East Anglia University of East Anglia Norwich United Kingdom; 5 Centre for Endocrinology William Harvey Research Institute, Barts and the London School of Medicine, Queen Mary University London United Kingdom; 6 Department of Paediatric Endocrinology The Children's Hospital at the Royal London Hospital Royal London Hospital London United Kingdom; 7 Medical Research Council Epidemiology Unit & Department of Paediatrics Institute of Metabolic Science, University of Cambridge Institute of Metabolic Science, University of Cambridge Cambridge United Kingdom; 8 Health Innovation East Health Innovation Cambridge United Kingdom; 9 Public Contributor public contributer Norwich United Kingdom; 10 Jenny Lind Children's Hospital Norfolk and Norwich University Hospital NHS Foundation Trust Norwich United Kingdom

**Keywords:** digital technology, health behavior change, obesity, young people, adolescents, participatory research

## Abstract

**Background:**

Specialist multidisciplinary clinics have been established to provide care for the burgeoning number of young people presenting with comorbidities related to severe obesity in childhood. Digital technology, an integral component of most young people’s lives, may enable clinics to offer accessible, ongoing support between appointments to the patients, thereby increasing the likelihood of successful health behavior change. However, while short-term engagement with technology-based behavior change interventions is good, engagement tends to decrease over time, limiting their overall impact. Little is known about the views of young people living with obesity on the role of digital technology as an adjunct to current traditional care pathways.

**Objective:**

This study aims to explore the views of adolescent patients and their families on whether digital technology should be used by obesity services to support health behavior change.

**Methods:**

Participants included patients aged between 10 and 16 years from an obesity clinic, along with their adult family members. Four focus groups and co-design workshops, facilitated by a cross-disciplinary team of clinicians, academics, and technology innovators, explored young people’s health priorities, identified the barriers to and facilitators of health behavior change, and co-designed ways in which technology could be used to support them in overcoming these barriers to achieving their health goals. Data were analyzed using inductive content analysis, with findings integrated with key co-design workshop outputs.

**Results:**

In total, 37 individuals participated, including 19 (51%) adolescents (n=11, 58% female) and 18 (49%) family members. The young participants, on average, were aged 13.4 (SD 1.68; range 10-16) years; the mean BMI was 36.6 (SD 0.3; range 34-47) kg/m^2^. The mean socioeconomic decile was 4.3 (SD 2; range 1-8). Participants did not mention weight as an important aspect of their health. Instead, mental health, sleep, and peer support were identified as the domains where patients felt they would most benefit from additional support. Addressing these aspects of health was viewed as foundational to all other aspects of health, with poor mental health, sleep, and social support reducing young people’s ability to engage in the process of health behavior change. Participants reported that technology could help provide this support as an adjunct to in-person support. Participants expressed a preference for technologies able to individually tailor content to the young person’s needs, including relatable peer-produced content. The need for support for both the young people and their family members was highlighted, along with the need to integrate in-person strategies to maintain engagement with any technological offering.

**Conclusions:**

There is clear potential for digital technology to support the holistic health priorities of young people receiving specialist care for the comorbidities of excess weight. This study’s findings will serve as a foundation for developing innovative approaches to the use of technology to support this high-need population.

## Introduction

### Background

The growing prevalence of childhood obesity is among the most important threats to public health globally [1]. In addition to being at a higher risk of significant childhood comorbidities [2], children living with overweight and obesity are more likely to experience obesity in adulthood [3]. Adults living with obesity are at markedly increased risk of a wide range of noncommunicable diseases, including cardiovascular diseases, diabetes, musculoskeletal disorders, and certain types of cancer [4].

In England, 22.7% of children aged 10 to 11 years were living with obesity in 2022 and 2023, with 5.7% classed as severely obese [5]. This equates to more than 2.5 million children eligible for specialist treatment according to the National Institute for Health and Care Excellence guidance [6]. Obesity currently costs the UK National Health Service (NHS) around £6 (US $8.1) billion annually, and, without a significant step-change in health intervention effectiveness, these costs are estimated to rise to over £9.7 (US $13.1) billion per year by 2050 [7].

Furthermore, childhood obesity is a significant factor in the maintenance of health inequalities [8]. Children growing up in the least economically advantaged neighborhoods are more than twice as likely to experience obesity and 4 times as likely to experience severe obesity as their peers growing up in the most advantaged neighborhoods [5]. In addition, obesity is more prevalent in children from Black and Asian (excluding Chinese) ethnic backgrounds than in children from White ethnic backgrounds [5].

The NHS Long Term Plan [9] is committed to improving the care available to children and young people experiencing health complications related to excess weight. As part of this commitment, 21 new Complications of Excess Weight (CEW) clinics located across England were commissioned in 2021 [10]. CEW clinics provide specialist biopsychosocial care, delivered by a multidisciplinary team comprising consultants, nurses, and allied health professionals (eg, dietitian, physiotherapist, psychologist, family therapist, and social worker). Given the limited evidence base for what constitutes optimal care for young people experiencing severe obesity, a key objective of the pilot clinics was to contribute to generating evidence on the most promising interventions and service models for this cohort.

During their treatment, young people under the care of a CEW clinic are encouraged to make multiple behavioral changes to improve their health, such as increasing activity, changing dietary patterns, and taking regular medication. However, adolescents face multiple barriers to successful and sustained behavioral change [11]. While CEW clinicians aim to support young people and their families to overcome these barriers, this support is only provided during scheduled clinic appointments.

Digital technology is now an integral component of nearly all young people’s lives [12]. Consequently, the increased use of digital technology may enable CEW clinics to offer accessible, ongoing support between appointments to their patients to increase the likelihood of successful health behavior change. Systematic reviews of technology-based interventions for overweight and obesity in adolescents have found some evidence of short-term improvement in dietary behaviors and physical activity [13] and significant reductions in BMI [14-16].

However, while short-term engagement with technology-based behavior change interventions is often good, this engagement tends to decrease over time [17-19], likely limiting their impact [20]. Furthermore, despite the acknowledged importance of involving intended end users in the development of digital health technologies [21], most currently available digital weight-management interventions were not developed with the involvement of patients or health care professionals [22].

### Objectives

This study aimed to explore the views of young patients and their family members on the potential for digital technology to enhance the support offered by CEW clinics to adolescents living with severe obesity. This study aimed to address the following research questions: (1) What are the most important unaddressed health priorities for adolescent CEW clinic patients from the perspective of patients and their family members? (2) What do adolescent CEW clinic patients and their family members perceive to be the key barriers to and facilitators of health behavior change, and to what extent do they believe that digital technology could assist in overcoming barriers? (3) What preferences do adolescent CEW clinic patients and their family members have for the design and delivery of digital technology to support health behavior change?

## Methods

### Design

**Figure 1:**
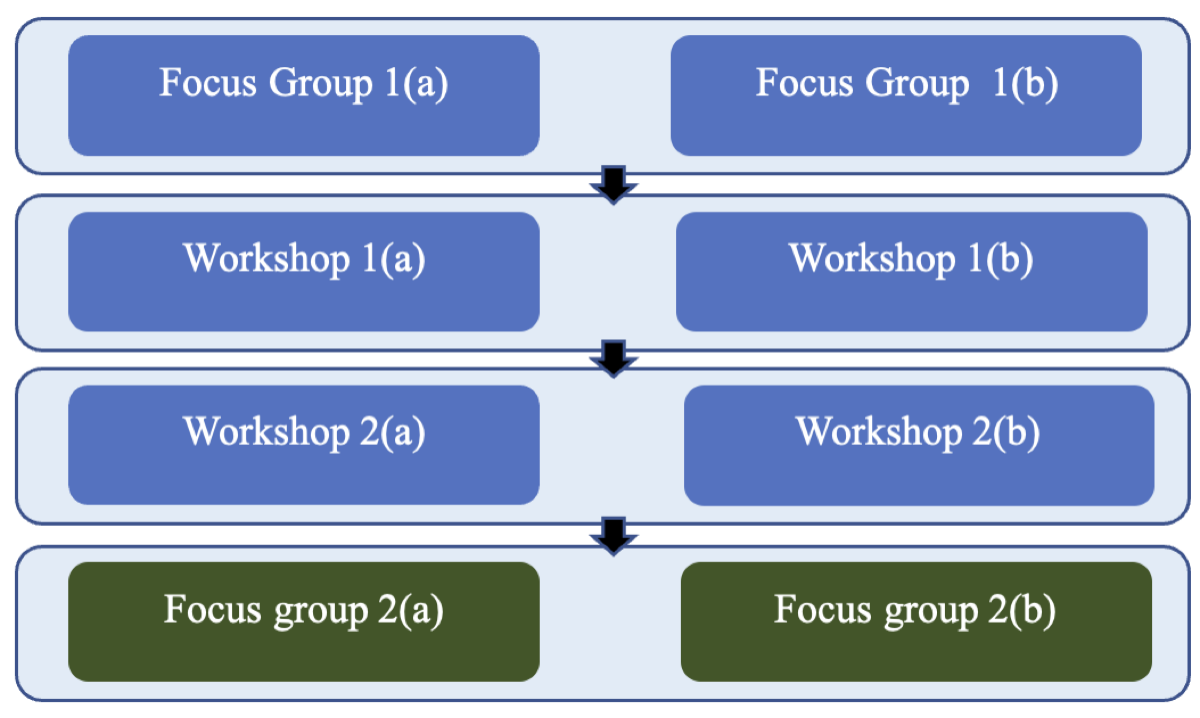
Study design schematic for a user-centered design study of digital technology to support young people being treated for obesity. (A) Focus groups and workshops shown in blue were conducted with the first participant group; (B) those shown in green were conducted with the second participant group.

This study used a participatory design involving a series of focus groups and co-design workshops (Figure 1) with adolescent CEW clinic patients and their adult family members. Each focus group and workshop was conducted twice on the same day to facilitate a larger number of families to participate while maintaining small group sizes (maximum 5 young people and 5 parents or guardians) to encourage participants to feel comfortable sharing their views openly. On the basis of feedback from potential participants and our young advisor, adolescents attended sessions together with their family members to help increase their confidence to participate. However, due to the potential for the presence of adult family members to inhibit the views young people were willing to share (and vice versa), participants were invited to break into separate groups during parts of the later sessions.

Focus group 1 and workshops 1 and 2 were conducted as an iterative process, with analysis of session 1 being used to inform discussion in the subsequent workshops. The same group of families participated in the first 3 sessions (focus group 1 and workshops 1 and 2). The final focus groups (focus group 2) reviewed the conclusions of focus group 1 and workshops 1 and 2. For the final focus groups, a different group of young people was recruited.

All focus groups and workshops were carried out face-to-face at nonclinical venues to maximize engagement. Each session lasted approximately 90 minutes. The study was carried out over a 12-month period from January to December 2023.

### Ethical Considerations

This study was approved by the NHS Health Research Authority following a favorable ethical opinion from the Wales Research Ethics Committee 4 (22/WA/0340). Informed consent (or informed parental consent and the young person’s assent in the case of those aged <16 y) was obtained and documented before participation in the study. To maintain confidentiality, each participant was assigned a numerical code. Each family was offered £50 (US $67.50) in high street shopping vouchers per focus group or workshop they attended and reimbursement of any travel expenses in recognition of the time and effort involved in their participation.

### Setting and Participants

The Healthy Futures clinic at Norfolk and Norwich University Hospital (NNUH) was established in 2022 as 1 of the 21 pilot CEW clinics commissioned by NHS England. The clinic provides specialist medical, dietetic, psychological, and family support to approximately 100 new patients each year. Children and young people eligible for the service are aged between 2 and 17 years, have a BMI 3.5 SDs above the mean, and have a suspected or diagnosed physical (eg, hypertension, joint or mobility problems, abnormal glucose metabolism, fatty liver disease, and sleep apnea) or psychological comorbidity.

Young people eligible to participate were patients of the NNUH CEW clinic aged between 10 and 16 years (N=67), who were willing and able to provide informed consent or assent to participate (and for those aged <16 y, whose parent or carer was willing and able to provide informed parental consent for their participation). Participating family members were consenting adult caregivers (aged >18 y) of eligible young participants, largely parents or carers, and also included a small number of other relatives (ie, grandparents and adult siblings).

All eligible families under the care of the NNUH CEW clinic at the time of recruitment were invited to participate via letter, followed up by conversations in face-to-face clinic appointments held in the pediatric department at NNUH. Participants were informed that the research was being conducted by a team of clinical and academic researchers in partnership with a technology company with the aim of improving the support offered by the Healthy Futures Clinic and other similar services.

As the study used a qualitative methodology, a formal sample size calculation was not appropriate. Instead, the sample size was selected based on empirical guidance regarding the number of focus groups and participants needed for a high likelihood of reaching theoretical saturation [23] while also taking into account pragmatic considerations such as the time frame of the study and resources available.

### Focus Groups

Focus groups were facilitated by authors BG (female) and BT (female), clinical researchers (both holding PhDs) experienced in facilitating focus groups regarding sensitive topics with young people and their families. In addition, one or more members of the CEW clinical team were present at each focus group and available to support participants. Focus groups were semistructured with content guided by approved topic guides, supported by visual aids presented via a slide deck. Each participant was provided with a notepad and encouraged to write or draw their ideas if they felt more comfortable communicating their views in this way than contributing verbally.

Topic guides were informed by the capability, opportunity, motivation, and behavior (COM-B) model of behavior change [24], which identifies 3 factors—capability, opportunity, and motivation—as necessary for successful behavior change. Topics covered in focus group 1 included (1) participants’ understanding of what “living a healthier life” meant, (2) perceived barriers to and facilitators of making changes toward a healthier life, and (3) what additional support participants believed young people accessing a CEW clinic would benefit from to help them make changes toward a healthier life (including the potential role of technology in providing this support).

Topics covered in focus group 2 included (1) the extent to which participants agreed with the health domains prioritized in previous focus groups and workshops as the most important unaddressed needs of CEW clinic patients, (2) participants’ views of existing digital health technologies designed to support the health domains prioritized in previous focus groups and workshops, and (3) participants’ views of existing health technology platforms through which young people accessing a CEW clinic could be provided with access to additional digital support. The technology platforms presented were chosen based on the priorities young people had highlighted in the preceding focus groups and workshops.

All focus groups were audio recorded and transcribed by members of the research team. All written or drawn contributions were collected and transcribed or photographed.

### Co-Design Workshops

Creative co-design workshops were facilitated by authors MF (male) and VF (female), experienced technology design professionals at LovedBy with previous experience of working in collaboration with adolescents to co-design digital health behavior change interventions. In addition, as with the focus groups, one or more members of the CEW clinical team were present at each workshop to support participants as required.

Workshop content was designed to encompass the first 2 stages of the Design Council’s Double Diamond design process [25]: the “discover” phase, involving gaining a detailed understanding of the design problem to be addressed through close engagement with the experiences and views of those impacted, and the “define” phase, involving using the insights gathered during the discovery phase to clearly define the design challenge and develop hypothesized solutions to be tested and refined in future phases. Activities involved emotional journey mapping of the young person’s day, paper-based cocreation exercises, initial prototyping of ideas generated, and gathering feedback on potential design solutions.

Due to the creative and nonlinear nature of the workshops, these were not audio recorded. Instead, the research team made observational field notes to capture key discussions and collected or photographed all written and visual outputs (ie, notes, drawings, and diagrams) produced by participants during the sessions.

### Data Analysis

As co-design involves collaborative real-time identification of challenges and cocreation of solutions between workshop participants and researchers [26], analysis in co-design is not a discrete process occurring after data collection is complete. Instead, initial phases of analysis take place concurrently with data generation through a collaborative process of participants and researchers working together to agree on the focus and outputs of the co-design process [27]. For instance, in workshop 1, the emotional journey mapping exercise was used to generate potential health domains to focus on, which were then discussed and prioritized by participants. In workshop 2, prototype design solutions based on ideas codeveloped in workshop 1 were collaboratively reviewed and refined to generate new insights into participant preferences for design and delivery of digital health technologies.

Analysis of the focus groups followed more conventional qualitative data analysis procedures. This involved inductive content analysis [28,29] of focus group transcripts (by BT and BG), involving data familiarization, iterative coding, and creating and refining content categories and subcategories.

Following the completion of the co-design process and inductive content analysis, the workshop outputs and focus group findings were reviewed and synthesized to generate overall answers to each of the study’s research questions. This process included mapping findings to the COM-B model and the theoretical domains framework of behavior change techniques [30] to support identification of potential intervention components.

### Patient and Public Involvement

A young person with personal experience of being supported by a CEW clinic acted as a young advisor and was an integral member of the study team. SW was involved in developing the recruitment strategy, advised on topic guides and workshop schedules, attended focus groups and workshops, and contributed to the interpretation and dissemination of findings.

## Results

### Overview

In total, 19 young people and 18 family members (n=37) took part in the study; 25 (68%) in the first set of focus groups and workshops 13 (35%) in the final focus groups (one young person was part of both cohorts). Across both cohorts, on average, the young participants were aged 13.4 (SD 1.68; range 10-16) years; mean BMI was 36.6 (SD 0.3; range 34-47) kg/m^2^. Of the 19 young people, 11 (58%) were female and 8 (42%) were male, and all were White except for 1 (5%) participant who identified as Asian. The demographic characteristics of participating young people and the wider patient cohort are presented in Table 1. On the basis of participants’ home postcodes, the mean socioeconomic decile was 4.3 (SD 2; range 1-8); 7 participants and their parents or guardians came from the lowest 3 socioeconomic deciles. No demographic data were collected for family members.

**Table 1:** Demographic characteristics of young people who participated in the user-centered design study and the wider cohort of patients under the care of Norfolk and Norwich University Hospital, Complications of Excess Weight clinic.

Characteristic	Study participants (n=19)	Wider patient cohort (n=48)
Age (y), mean (SD; range)	13.4 (1.68; 10-16)	13.6 (1.6; 10.2-15.8)
BMI (kg/m^2^), mean (SD; range)	36.6 (0.3; 34-47)	40 (0.3; 32.5-50)
Female sex, n (%)	11 (58)	22 (46)

### Research Question 1: What Are the Most Important Unaddressed Health Priorities for Adolescent CEW Clinic Patients and Their Family Members?

When asked to define what living a “healthy life” meant to them, participants’ responses fell into 3 categories: achieving good mental health, implementing healthier behavior patterns, and having positive health outcomes. The category of achieving good mental health encompassed feeling happy and “good in yourself” as well as feeling confident and satisfied with their appearance. Health behaviors viewed as important to living a healthier life included eating a healthy diet, incorporating exercise or other enjoyable activities into their daily lives, and regularly getting enough sleep. Participants mentioned wanting to achieve health outcomes such as having more energy and living longer. Participants did not mention losing weight or maintaining a healthy weight as an important aspect of health, except as it related to their mental health, ability to make health behavior changes, or improve their health outcomes.

Three domains emerged as the most important health priorities for both young people and their family members (areas for which adequate support is currently lacking): mental health, sleep, and peer support.

Mental health was viewed as the most important unaddressed health priority by nearly all young people and their family members. The mental health support currently available was often viewed as inadequate due to difficulties accessing specialist mental health services and long waitlists for treatment:

Persistent [mental health] problems I like wrote about and described to like doctors for years, nothing happens because the waitlists are that long.... I think it’s the thing I’d like to see the most done about, because there seems to be the worst area in my experience.

Young person

She’s had two referrals to CAMHS [child and adolescent mental health services] and they’ve both been rejected both times. And they’re when we’ve been right at the worst moments as well. And that’s where you need the support.

Family member

Participants spoke about schools as often being the only source of mental health support available to them. The quality of the mental health support provided by schools was described by participants as highly variable. Some families were highly satisfied with the support they had received from their child’s school. However, others were less than satisfied with the support available; several participants reported that the responsibility for pastoral support in their school fell to staff with little training in mental health who were therefore ill-equipped to provide the specialist support they or their child needed.

Mental health was viewed as foundational to all other aspects of health, with poor mental health reducing the young people’s ability to engage in the process of health behavior change:

Well for me mental your mental health is key, isn’t it? You’re happy. Everything else falls into place. Then it’s come to light that if your if your mental health isn’t great, and then everything else drops off, you know.

Family member

In addition, sleep was viewed as an important area of unmet need by some young people. This emerged particularly strongly in the emotional journey mapping activity (Multimedia Appendix 1) during which young people and their family members highlighted the impact of poor-quality sleep on the young person’s daytime mood, energy, and functioning. The young person’s mood and ability to function (for instance, to attend school regularly and on time), in turn, impacted the well-being of the wider family. None of the participants reported having previously received professional support focused specifically on sleep, but some had tried using self-help resources with varied success:

She found it so difficult to sleep. We’ve tried all sorts, at the minute she’s listening to Whale noises and stuff like that just to try and block everything else what’s going on in her head to go to sleep. But even then, sometimes that’s not working and it’s difficult for her. And then when it comes to school, it’s like “I can’t go to school. I don’t wanna go to school.”

Family member

The final area of unmet need highlighted by participants was peer support. Peer support was seen as an important factor in helping young people successfully change their behaviors by nearly all adult family members; good peer support was also seen as a protective factor for mental health. Young people were more divided on the importance of this area depending on the quality of their existing friendships. Some young people were satisfied with the support provided by their existing friendships and therefore did not view peer support as something from which they would benefit:

I’ve always had like really helping like relationships with my friends and my family and that...if I didn’t have friends, I don’t know how will it feel.

Young person

However, others described having few friendships and finding it hard to relate to peers:

I’ve had worse than everyone who I’ve been in my school with, so there’s no point in asking how they feel.

Young person

Experiences of bullying and neurodiversity were viewed as key barriers to young people forming supportive peer networks.

The opportunity to meet people facing similar challenges was viewed as being potentially beneficial both for the young people themselves and their family members:

At school I think young people are so judged by what they look like. And I think that [opportunities for peer support] would kind of make them not feel as bad. Yeah, if you know what I mean. Because they’ve got the support, and they’ll realise that they’re not the only one who struggles.

Family member

Adult participants reported that they valued the opportunity to do this during the focus groups and workshops and would appreciate further opportunities to connect with parents and carers in similar situations to themselves.

### Research Question 2: What Do Adolescent CEW Clinic Patients and Their Family Members Perceive to Be the Key Barriers to and Facilitators of Health Behavior Change, and to What Extent Do They Believe That Digital Technology Could Assist in Overcoming Barriers?

Poor mental health was cited as a significant barrier to health behavior change. Anxiety related to social interactions and engagement in group health activities, sometimes related to previous experience of bullying or low self-esteem, was viewed as particularly problematic. Poor sleep was also noted as impacting motivation to engage in health behavior change. Disruption to routines, for instance, due to school holidays, and seasonal pressures, such as Christmas, Easter, and summer breaks, were viewed as making it more difficult to maintain healthy behaviors. In addition, financial considerations were important barriers to health behavior change for many families. The high cost of healthy food and activities and limited access to affordable transport, particularly for those living in rural areas, were the main financial barriers identified by the participants:

I was trying to find, you know, the money to go and do lots of [active] things, do those things. Time is not an issue...I can give her the time that she needs. Yeah, it’s whether I’ve got the funds to do it.

Family member

Social support was viewed as the most important facilitator of health behavior change. The support and encouragement of family were most often seen as imperative, with changes undertaken as a family considered easier to maintain than those made by the young person in isolation:

They [family] can encourage you to go out and do like exercise and things like that, and encourage you to eat healthier options, but also they kind of do it as well with you.

Young person

Support from peers and school staff was also an important facilitator of making and maintaining changes for some participants.

Digital technology was viewed both as a barrier to and a potential facilitator of health behavior change. The opportunity costs imposed by intensive use of digital technology were seen as a barrier to health behavior change, with time spent engaging with screen-based activities (eg, playing video games, using social media, and watching online content) detracting from the time available for physical activity and encouraging snacking behavior:

It makes one want to eat as well, you’re sat there doing nothing, well looking at screen, you want, yeah, they, they do because...it’s like “oh, I’m sitting here, I can just snack on something.” Whereas if they’re out and about, they’re not thinking about it.

Family member

The content young people were exposed to online was also sometimes viewed as creating barriers to health behavior change. For instance, seeing images of “ultrafit” celebrities and influencers on social media was viewed as creating unrealistic expectations, impacting motivation to change behaviors.

Many young people reported having used health apps, for instance, step trackers or food diaries, and found these helpful, at least to some extent. However, adult family members spoke about the costs associated with some health technologies, creating barriers to sustained use, for instance, health apps that charged a monthly subscription after an initial free trial period:

Even working families struggling and even having to buy an app, that’s maybe only £4.99 but that’s £4.99 that’s coming out of the food bill.

Family member

Some aspects of social media were also viewed as facilitators of health behavior change both by young people and their family members. Helpful online content that the families spoke about included inspiring stories of health behavior change, ideas for healthy recipes, and suggestions for enjoyable movement, particularly activities and challenges that could be completed together as a family.

### Research Question 3: What Preferences Do Adolescent CEW Clinic Patients and Their Family Members Have for the Design and Delivery of Digital Technology to Support Health Behavior Change?

Extensive use of digital technology was reported to be a universal aspect of the lives of all the young participants. As such, support delivered or facilitated via technology was viewed as a potentially useful adjunct to face-to-face support. However, participants were also clear that it should not be a replacement for the support currently available or indeed seen as a substitute for increasing the availability of face-to-face support in areas of unmet need.

Key perceived benefits of using digital technology to provide support included more immediate access than face-to-face support and increased privacy and autonomy for young people in relation to the support they receive:

They can dip in and out of that they can actually access for themselves, yeah, when they need it, when they want it and nobody else knows what they’re doing.

Family member

I know that [being able to access support via technology] would help me and I know it would definitely help a lot of my friends that can’t speak out, and their friends and their like parents won’t give them that support.

Young person

Participants viewed digital technology as having the potential to help address all 3 of the unmet health priorities identified: mental health, sleep, and peer support. The privacy and autonomy offered by digital interventions were viewed as particularly valuable in areas that might be more sensitive, such as mental health. Technology was also seen as a practical way of bringing people with similar experiences together to provide peer support and build a sense of community. In addition to facilitating direct content sharing with other families, participants expressed that they would value having access to digital content featuring others who have been through similar experiences. Peer-led content was seen as more relatable than other digital content, lessening feelings of isolation and boosting motivation.

Participants expressed largely positive views of the examples they were shown of existing digital health technologies designed to support mental health (Lumi Nova) [31], sleep (digital cognitive behavioral therapy [Sleepio] [32] for insomnia), and peer support (Kooth) [33]. Suggested adaptations to the example technologies were largely related to the needs or preferences of the specific young person (eg, to make the technology more appropriate for their age range) rather than CEW clinic users more widely.

Most participants were not previously aware of the example technologies discussed, and parents and carers reported that they often had difficulty finding out about the resources available to them. Participants felt that there is a need for a platform bringing together available resources in an easily accessible format. They expressed a preference for a platform that is appealing and appropriate for the targeted age range and able to tailor content to the specific health needs of individual young people, including relatable peer-produced content.

Participants expressed the need to maintain the young person’s motivation to engage with a digital platform. They suggested that this could be achieved through integration with online applications the young person already uses frequently or through support from family members and clinicians. Family members felt that they would also benefit from access to digital resources and communities that could help them support the young person.

During data analysis, concepts were mapped under the components of the COM-B model and the constructs of the theoretical domains framework. Behavior change techniques were identified to support future work in the development of intervention components. The concepts mapped under these models and the behavior change techniques identified are presented in Multimedia Appendix 2.

SW is a coauthor of this paper, and her reflections on being involved in the study are as follows:

I was interested in getting involved in the project as I need to make some changes to my own health habits. As a young person I think it’s a very important project as access to good and accurate advice about health and lifestyle can be hard and daunting. There is often conflicting advice online and things aren’t always presented in a way that makes them accessible. It’s also not always that young people don’t know the information, but that they don’t know how to act on it. Particularly if there are circumstances outside their control such as living or financial situation. I know people say it doesn’t cost money to be healthy but it can definitely help make it easier.

Also, as someone who enjoys programming, the technical side of the project interested me to see how an app could be used and how interactive it could be. During the focus groups I found hearing several young people’s perspectives interesting. We had different views sometimes on who would be best to provide information and it was good to discuss our ideas and work together to think about what would have the broadest appeal to as many young people as possible.

I think it’s important to understand that young people have access to lots of information and that this project needed to focus on what will really draw young people to make changes. For me personally, I would find it most engaging to hear from professionals in short videos/blogs about different topics. I also especially thought the close work with the company, LovedBy, was helpful as they listened to our thoughts and then helped us shape them to what was possible technically.

The challenges I saw with the project were drawing enough information from participants in a short time scale and it would be really good to have follow up sessions even if online as I think people often think of things after the event that would be useful. Success to me on this project would be a resource for young people to find good and accurate information about getting and maintaining a healthy lifestyle. Real advice but with thought about how lifestyles are different for young people depending on their circumstances. Advice on how to make the healthier habits both realistic but also interesting to achieve would be good. Also, a way to set goals and measure achievements within an app/site would encourage people with forming better habits.

## Discussion

### Principal Findings and Comparison With Prior Work

Digital technology offers the potential to provide young people with severe obesity with access to additional support between CEW clinic appointments to increase the likelihood of successful health behavior change. This study aimed to explore the views of young CEW clinic patients and their family members on the use of digital technology to support them in achieving their health priorities.

Young people and their family members were supportive of the idea of increased use of digital technology by CEW clinics to facilitate access to additional support to address currently unmet needs. However, participants expressed the view that this support should be an adjunct to, not a replacement for, face-to-face support provided by clinicians. This view aligns with the evidence base, as there is currently little evidence for the effectiveness of digital interventions for pediatric obesity as stand-alone interventions outside of a comprehensive package of support [15,34].

When participants talked about health, they did not focus on behavior change that directly affected their weight but on changes that would help them feel better and would indirectly enable them to engage with behavior change. We believe this interesting finding was made possible by the nondirective, participant-led approach adopted in this study. This insight should shape the content of any digital intervention for adolescents under the care of a CEW clinic, ensuring adequate focus on concerns beyond weight to maximize young people’s engagement.

Participants identified 3 domains in which they would particularly benefit from increased support: mental health, sleep, and peer support. Improved mental health, sleep, and increased social support (both from peers and family members) were seen as facilitators of wider health behavior change as well as overcoming financial barriers and balancing the benefits of technology against the negative impacts of excessive screen time. Participants identified several ways in which the use of digital technology could be a barrier to health behavior change, including exposure to harmful online content and excessive screen time promoting sedentary behavior and snacking. Care must be taken to minimize these potential negative effects when developing digital technology for this patient group. Many of the barriers to and facilitators of health behavior change identified by participants in this study overlap with those identified in a Canadian study of adolescents living with severe obesity receiving multidisciplinary clinical care [35]. This study highlighted 3 factors that impact behavior change across lifestyle domains: perceived controllability, the impact of mental health, and social relationships and interactions. In line with this study, the Canadian team identified both mental health and sleep as important priority areas for young people living with obesity and concluded that “lifestyle-based interventions for behavior change should evolve to emphasize outcomes beyond weight status to include mental health as a primary intervention focus and outcome.”

All 3 domains prioritized by the participants in our study as unmet needs were areas where technology has previously been found to positively impact clinical outcomes. Kooth provides a web-based service that gives children and young people access to an online community of peers and experienced counselors [33]. Accessing Kooth is associated with reductions in psychological distress, suicidal ideation, loneliness, and reported self-harm. Lumi Nova is a mobile app that has been shown to reduce anxiety through the delivery of exposure-based cognitive behavioral therapy strategies via immersive gaming technology [31]. Sleepio is a cognitive behavioral therapy program recommended by the National Institute for Health and Care Excellence for improving sleep and can be accessed through a website or an app [32].

These existing digital interventions were viewed as potentially helpful, but current awareness and the use of digital resources were low. Participants expressed enthusiasm for the creation of a platform bringing together digital resources tailored to the needs of individual young people and their family members, including content created by peers. The use of digital technology to connect young patients and their family members with others with similar experiences was also supported.

In line with previous research [11,16,36], the findings of this study make the role of family support in the success or failure of adolescents’ health behavior change efforts clear. As such, any digital technology developed in this area must take into account the needs and preferences of not only the young patient but also their wider family support network. For instance, a digital intervention could include a forum for parents and carers to share ideas and seek support from both professionals and peers and include lifestyle interventions aimed at the family unit. Young people expressed enthusiasm for activities and challenges aimed at the whole family, suggesting this approach is likely to be more acceptable than an intervention targeting the young patient alone.

### Strengths and Limitations

To the best of our knowledge, this is the first study to seek the views of adolescents under the care of a specialist pediatric obesity clinic and their adult family members regarding the use of digital technology to support health behavior change. We were able to engage with a diverse range of family characteristics, accessing the clinic through a creative participatory design facilitated by a cross-disciplinary team of clinicians, academics, innovators, and a young service user. The benefits of involving the intended users of a health technology early in the design process are well established [37].

The limitation of this study is that it was conducted in a single site with patients from only 1 CEW clinic and with a self-selecting sample, possibly limiting the transferability of the findings. Furthermore, the geographical area in which this clinic is located (Norfolk, United Kingdom) has a population with limited ethnic diversity, which was reflected in the demographics of the sample recruited. However, aligned with the population profile of Norfolk, participating families were from diverse socioeconomic and urban-rural backgrounds. Given the higher prevalence of obesity in young people from non-White ethnic backgrounds [5], it will be important that future research explores the relevance of this study’s findings to families from a diverse range of cultural backgrounds. Multisite studies will likely be needed to adequately address this limitation in future research.

In addition, young people and their adult family members participated in the focus groups and workshops alongside one another. While this was intended to increase the confidence of young people to participate, and we believe it was successful in this regard, it may have inhibited some participants from both groups from expressing their views as openly as they would have if their family members had not been present. However, young people and family members participating together was also a benefit as it allowed both to respond to each other’s views and also allowed for family dynamics likely to be important to the use of digital interventions to be observed.

### Conclusions

This study highlights the potential for digital technology to be used to provide young people experiencing health complications related to obesity with additional support to make changes to improve their health. The findings suggest that such technology-based interventions should focus on outcomes beyond weight, provide individually tailored content, and support the needs of the wider family and not just the young patient alone to maximize their impact. Technological support should be offered as an adjunct to multidisciplinary, clinic-based interventions and may require ongoing clinician support to ensure engagement is maintained over time for potential benefits to be realized.

## Appendix

Multimedia Appendix 1 Digital representation of the output of an emotional journey mapping activity carried out in the first co-design workshop conducted as part of a user-centered design study of digital technology to support young people being treated for obesity.

Multimedia Appendix 2 Recommendations for future development of digital technology to support young people being treated for obesity based on the application of the capability, opportunity, motivation, and behavior model and the theoretical domains framework.

## Abbreviations

CEW: Complications of Excess Weight
COM-B: capability, opportunity, motivation, and behavior
NHS: National Health Service
NNUH: Norfolk and Norwich University Hospital

## Data Availability

The datasets generated or analyzed during this study are available from the corresponding author on reasonable request.
