# Supplementary figures and images for: Exploring the Use of Digital Technology to Support Health Behavior Change in Young People Under the Care of Complications of Excess Weight (CCEW) Clinics: Qualitative Patient-Centered Design Study
Source: JMIR Form Res. 2025 Oct 15;9:e64947. doi: 10.2196/64947 (PMC12572744; doi:10.2196/64947)

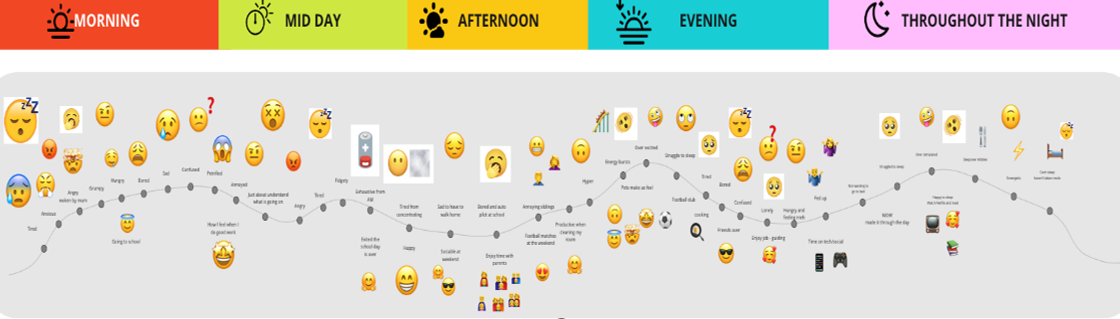

Supplement: Multimedia Appendix 1 [file formative_v9i1e64947_app1.png]
