# Supplementary material for: Exploring the Use of Digital Technology to Support Health Behavior Change in Young People Under the Care of Complications of Excess Weight (CCEW) Clinics: Qualitative Patient-Centered Design Study
Source: JMIR Form Res. 2025 Oct 15;9:e64947. doi: 10.2196/64947 (PMC12572744; doi:10.2196/64947)
Supplement: Multimedia Appendix 2 [file formative_v9i1e64947_app2.docx]

**Table S1.** Recommendations for future development of digital technology to support young people being treated for obesity based on the application of the capability, opportunity, motivation, and behavior (COM-B) model and the theoretical domains framework (TDF).

| Concept | COM-B | TDF | Suggested behavior change techniques |
| --- | --- | --- | --- |
| Happiness about weight and weight loss journey | - AM^a^ | - Emotions | - Reduce negative emotions (11.2) |
| Increasing confidence and their beliefs about being proud of themselves | - RM^b^ | - Beliefs about capabilities | - Verbal persuasion about capability (15.1) - Self-talk (15.4) |
| Encouraging positive body image and addressing body image | - RM - SO^c^ | - Beliefs about capabilities - Social identity - Social influence | - Verbal persuasion about capability (15.1) - Self-talk (15.4) - Social support (unspecified; 3.1) - Social support (professional; 3.2) - Social comparison (6.2) - Credible source (9.1) - Identity associated with changed behavior (13.5) |
| Addressing anxiety, depression, boredom, and stress | - AM | - Emotions | - Reduce negative emotions (11.2) |
| Dealing with peer pressure | - AM - SO - PsyC^d^ | - Emotions - Social influence - Behavioral regulations | - Reduce negative emotions (11.2) - Social support (unspecified; 3.1) - Social support (professional; 3.2) - Behavior substitution (8.2) |
| Addressing being the victim of bullying | - AM - SO | - Emotions - Social influence | - Reduce negative emotions (11.2) - Social support (unspecified; 3.1) - Social support (professional; 3.2) |
| Knowledge and consistent information about what a healthy life means: a balanced diet, exercise they enjoy, having a healthy sleep pattern, and being more energetic; education on healthy food options, portion size, and calories in and out | - PsyC - PhyC^e^ - RM | - Knowledge - Skills - Beliefs about capabilities | - Instruction on how to perform the behavior (4.1) - Information about antecedents (4.2) - Information about health consequences (5.1) - Instructions on how to perform the behavior (4.1) - Behavioral practice (8.1) - Graded tasks (8.7) - Verbal persuasion about capability (15.1) - Self-talk (15.4) |
| Addressing the need for professional support | - SO | - Social influence | - Social support (unspecified; 3.1) - Social support (professional; 3.2) |
| Addressing the need for group activities and online chat | - SO | - Social influence | - Social support (unspecified; 3.1) - Social support (professional; 3.2) |
| Education on what food to shop for and keep at home | - PO^f^ | - Environmental context and resources | - Social support (practical; 3.2) - Prompt (cues; 7.1) - Remove the aversive stimulus (7.5) - Restructuring the physical environment (12.1) - Restructuring the social environment (12.2) - Avoidance or reducing exposure to cues for the behavior (12.3) - Adding objects to the environment (12.5) |
| Support in building and maintaining a routine | - PsyC | - Behavioral regulation | - Self-monitoring of behavior (2.3) |
| Addressing the need for family support | - SO | - Social influence | - Social support (unspecified; 3.1) - Social support (professional; 3.2) |
| Addressing the need for exercise support | - PsyC - SO - AM | - Behavioral regulation - Social influence - Reinforcement | - Self-monitoring of behavior (2.3) - Social support (unspecified; 3.1) - Social support (professional; 3.2) - Nonspecific reward (10.3) - Social reward (10.4) |
| Addressing the role of seasonality (half term, Christmas, and Easter) | - PsyC - SO - RM | - Behavioral regulation - Social influence - Beliefs about capabilities - Beliefs about consequences | - Behavior substitution (8.2) - Social support (unspecified; 3.1) - Social support (professional; 3.2) - Problem-solving (1.2) - Instruction on how to perform behavior (4.1) - Information about health consequences (5.1) - Salience of consequences (5.2) - Anticipated regret (5.5) - Information about emotional consequences (5.6) - Pros and cons (9.2) - Comparative imagining of future outcomes (9.3) |
| Supporting setting realistic expectations | - PsyC | - Knowledge | - Instruction on how to perform a behavior (4.1) - Information about antecedents (4.2) - Information about health consequences (5.1) |

^a^AM: automatic motivation.

^b^RM: reflective motivation.

^c^SO: social opportunity.

^d^PsyC: psychological capability.

^e^PhyC: physical capability.

^f^PO: physical opportunity.
